# Supplementary material for: Multiplexed single-cell imaging reveals diverging subpopulations with distinct senescence phenotypes during long-term senescence induction
Source: GeroScience. 2025 Jan 23;47(3):3891–905. doi: 10.1007/s11357-024-01503-7 (PMC12181536; doi:10.1007/s11357-024-01503-7)
Supplement: Supplementary file 1 — Supplementary file1 (DOCX 16 KB) [file 11357_2024_1503_MOESM1_ESM.docx]

| **Target** | **Secondary Species** | **Catalog #** | **Dilution Used** |
| --- | --- | --- | --- |
| p53 | Mouse | CST 48818 | 1:400 |
| p21 | Goat | R&D AF1047 | 1:100 |
| p16 | Mouse | CINtec 705-4793 | ~1ug/mL Undiluted |
| p-p65 | Mouse | CST 3036 | 1:100 |
| PARP1 | Rabbit | AbClonal A0942 | 1:400 |
| GATA4 | Rabbit | AbClonal A13756 | 1:200 |
| JAK2 | Rabbit | Invitrogen 702434 | 1:250 |
| pSTAT3 | Mouse | CST 9145 | 1:100 |
| IL-6 | Goat | R&D AF206NA | 1:100 |
| RB | Mouse | CST 9309 | 1:500 |
| pRB | Rabbit | CST 9308 | 1:1000 |
| IL-8 | Mouse | R&D 6217 | 1:100 |
| p38 | Rabbit | CST 8690 | 1:100 |
| PLAUR | Rabbit | AbClonal 1397 | 1:400 |
| p65 | Rabbit | ProteinTech 10745-AP-1 | 1:200 |
| p-p38 | Mouse | CST 9216 | 1:200 |
| STAT5 | Rabbit | AbClonal AP0887 | 1:250 |

Supplemental Table 1. List of Antibodies used, including dilution factor used and

catalog number
