# Supplementary figures and images for: Multiplexed single-cell imaging reveals diverging subpopulations with distinct senescence phenotypes during long-term senescence induction
Source: GeroScience. 2025 Jan 23;47(3):3891–905. doi: 10.1007/s11357-024-01503-7 (PMC12181536; doi:10.1007/s11357-024-01503-7)

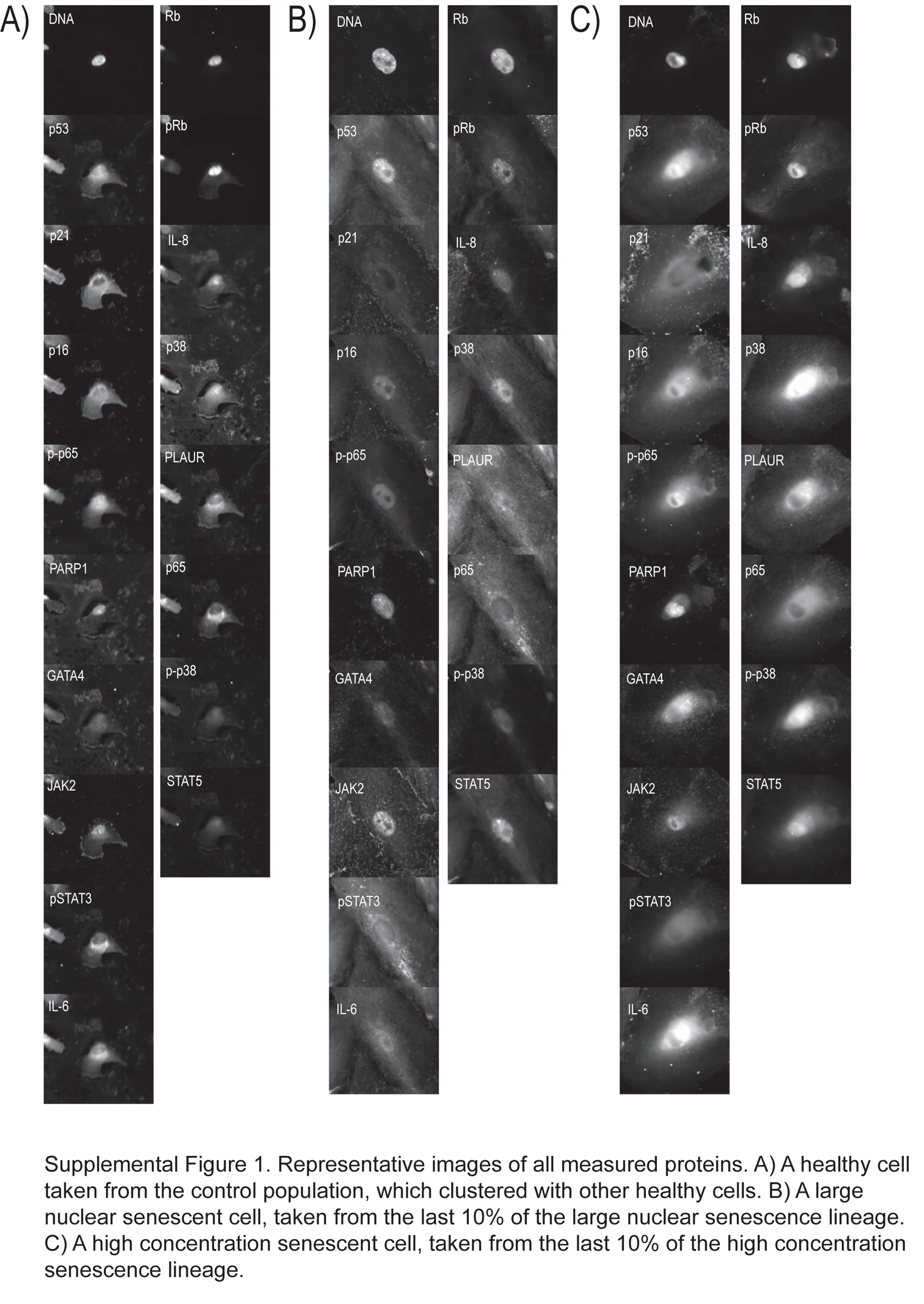

Supplement: Supplementary file 2 — Supplementary Fig. 1 [file 11357_2024_1503_Fig8_ESM.png]

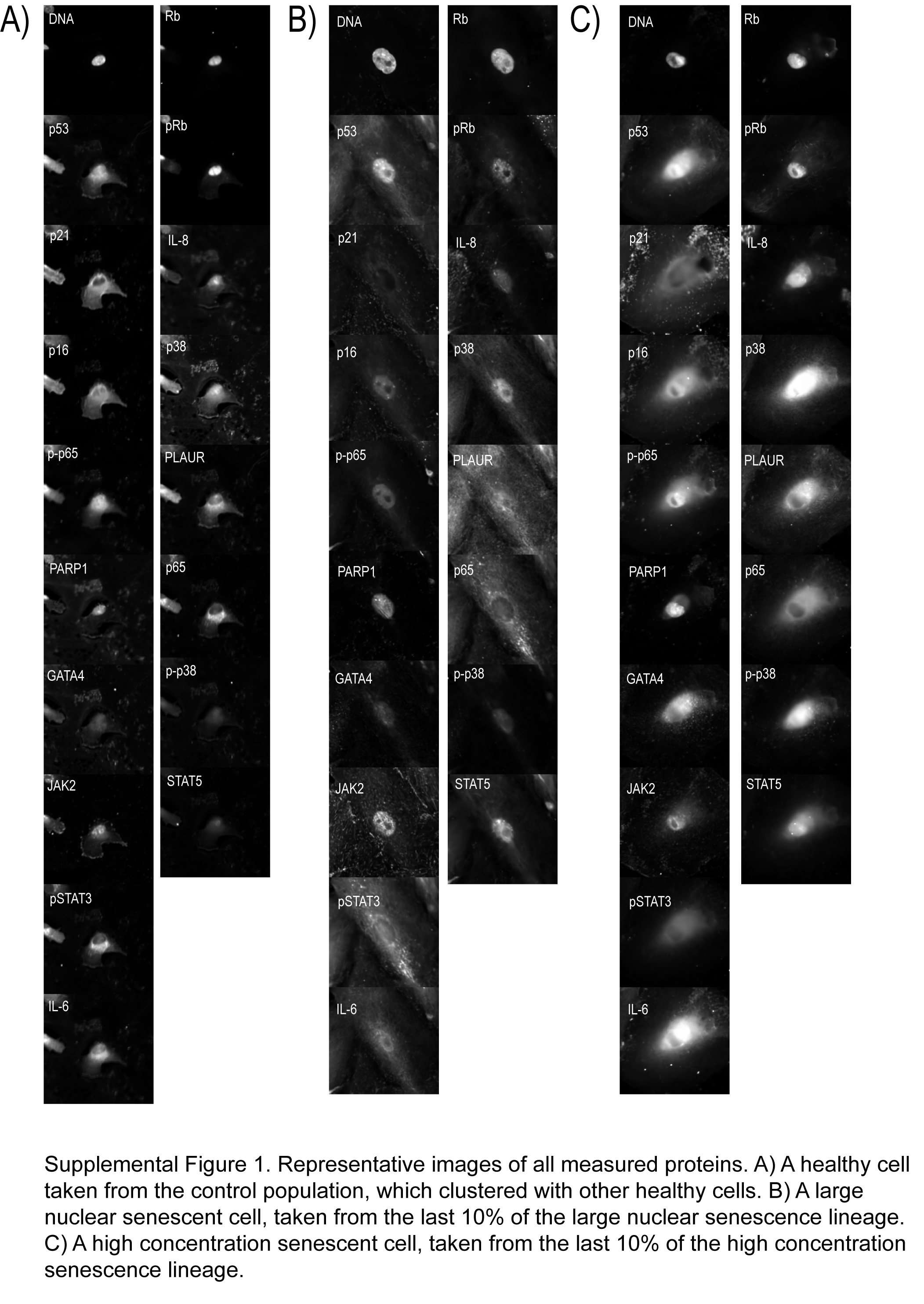

Supplement: Supplementary file 3 — High resolution image (TIF 5903 KB) [file 11357_2024_1503_MOESM2_ESM.tif]

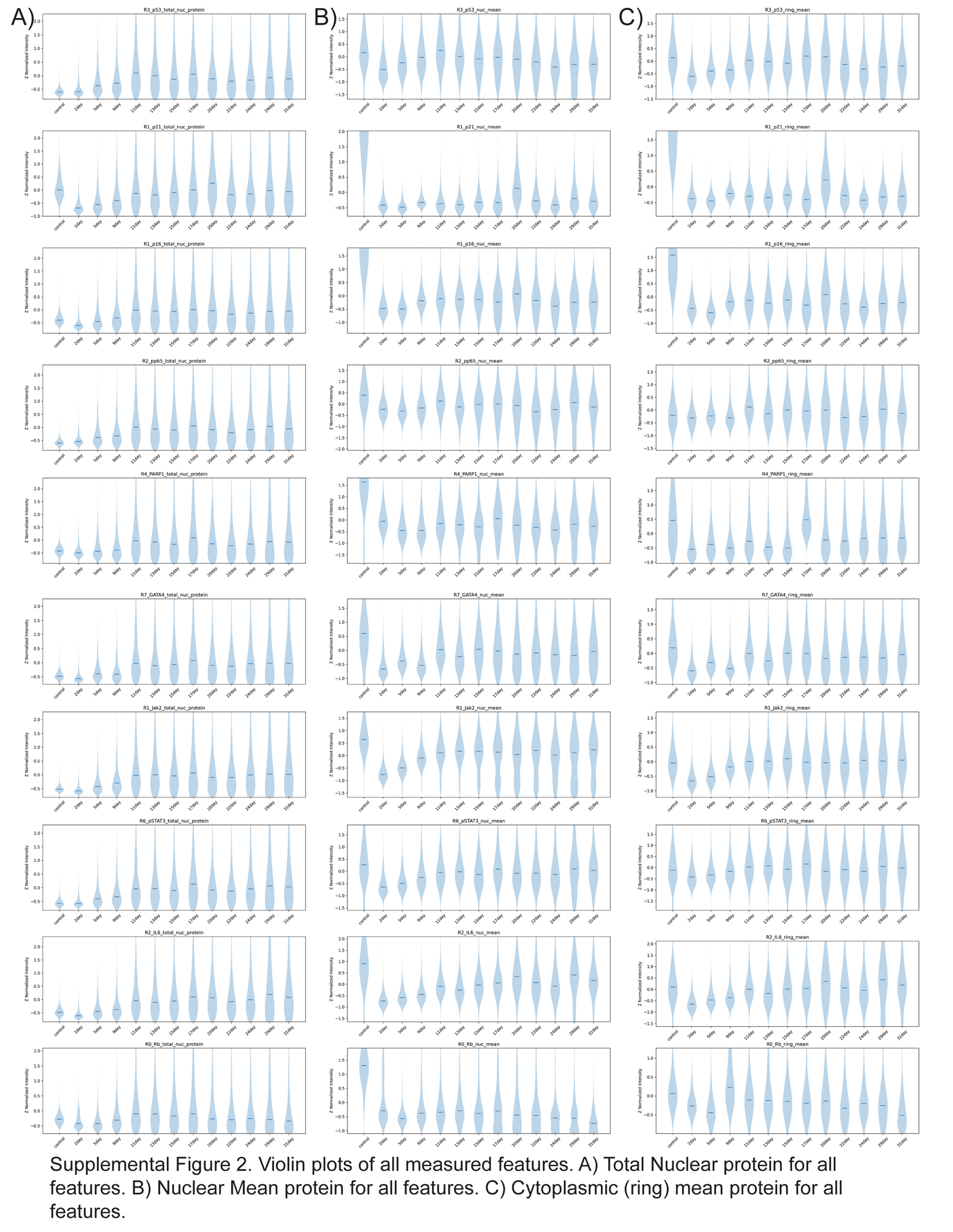

Supplement: Supplementary file 4 — Supplementary Fig. 2.1 [file 11357_2024_1503_Fig9_ESM.png]

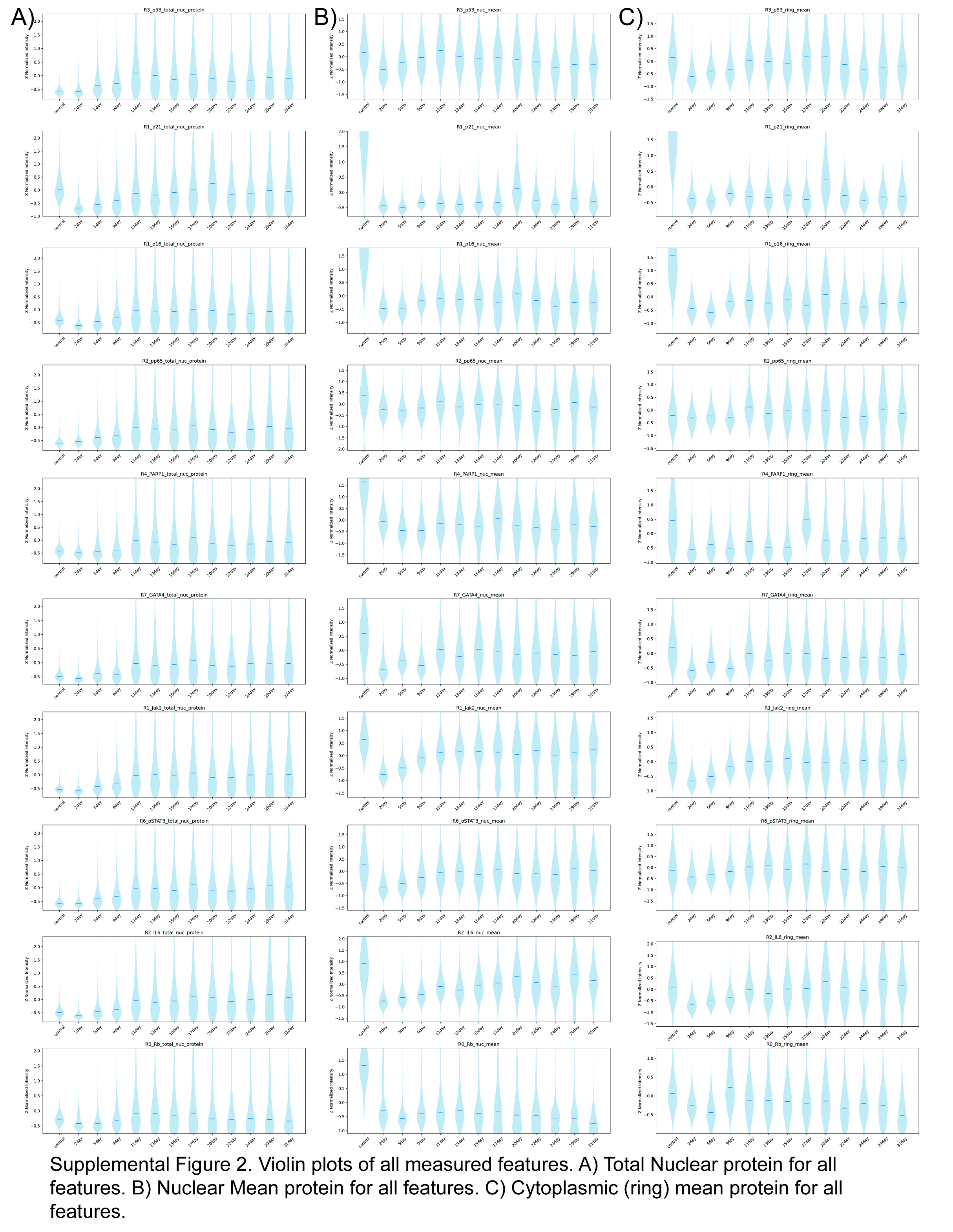

Supplement: Supplementary file 5 — High resolution image (TIF 2126 KB) [file 11357_2024_1503_MOESM3_ESM.tif]

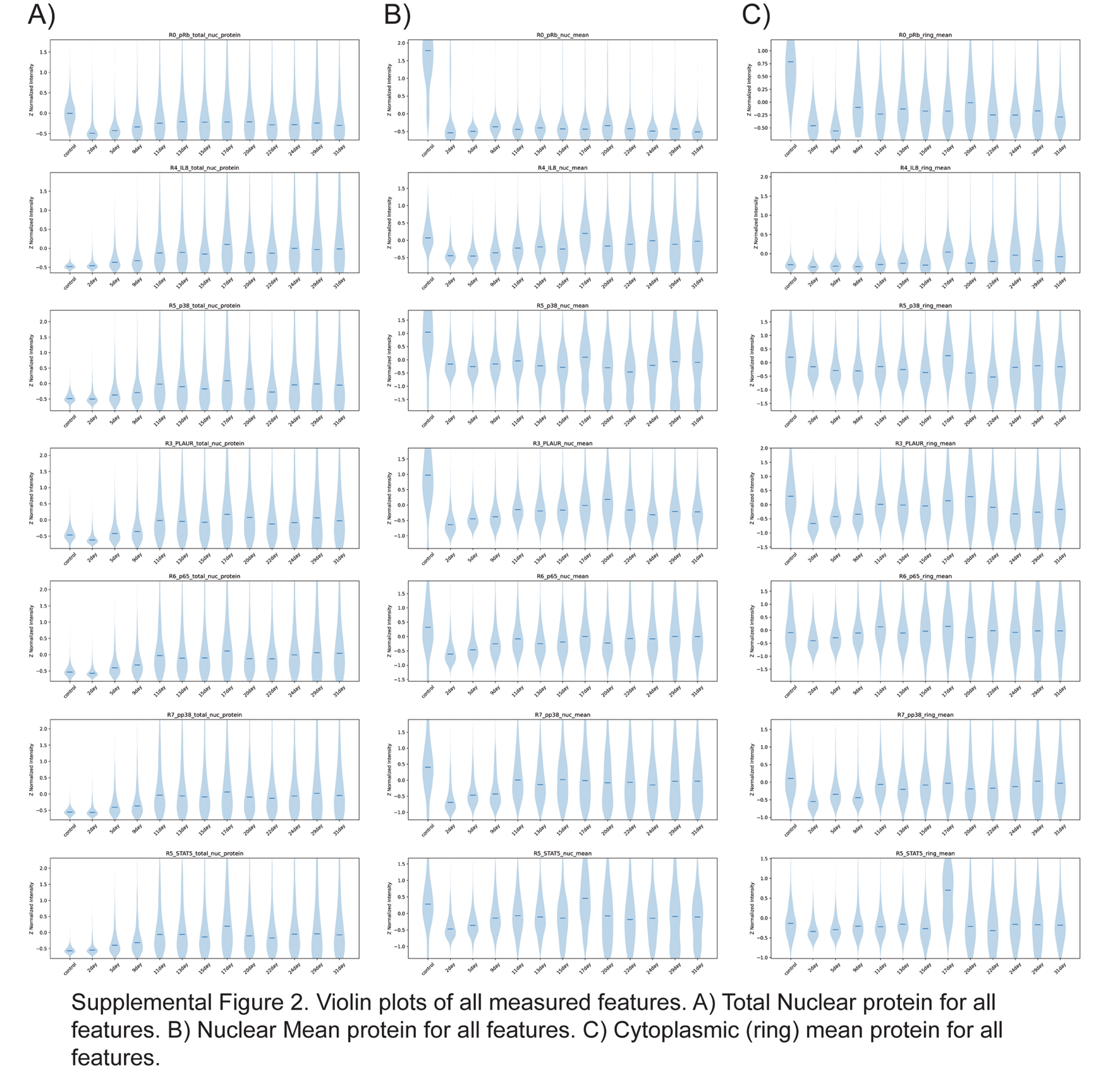

Supplement: Supplementary file 6 — Supplementary Fig. 2.2 [file 11357_2024_1503_Fig10_ESM.png]

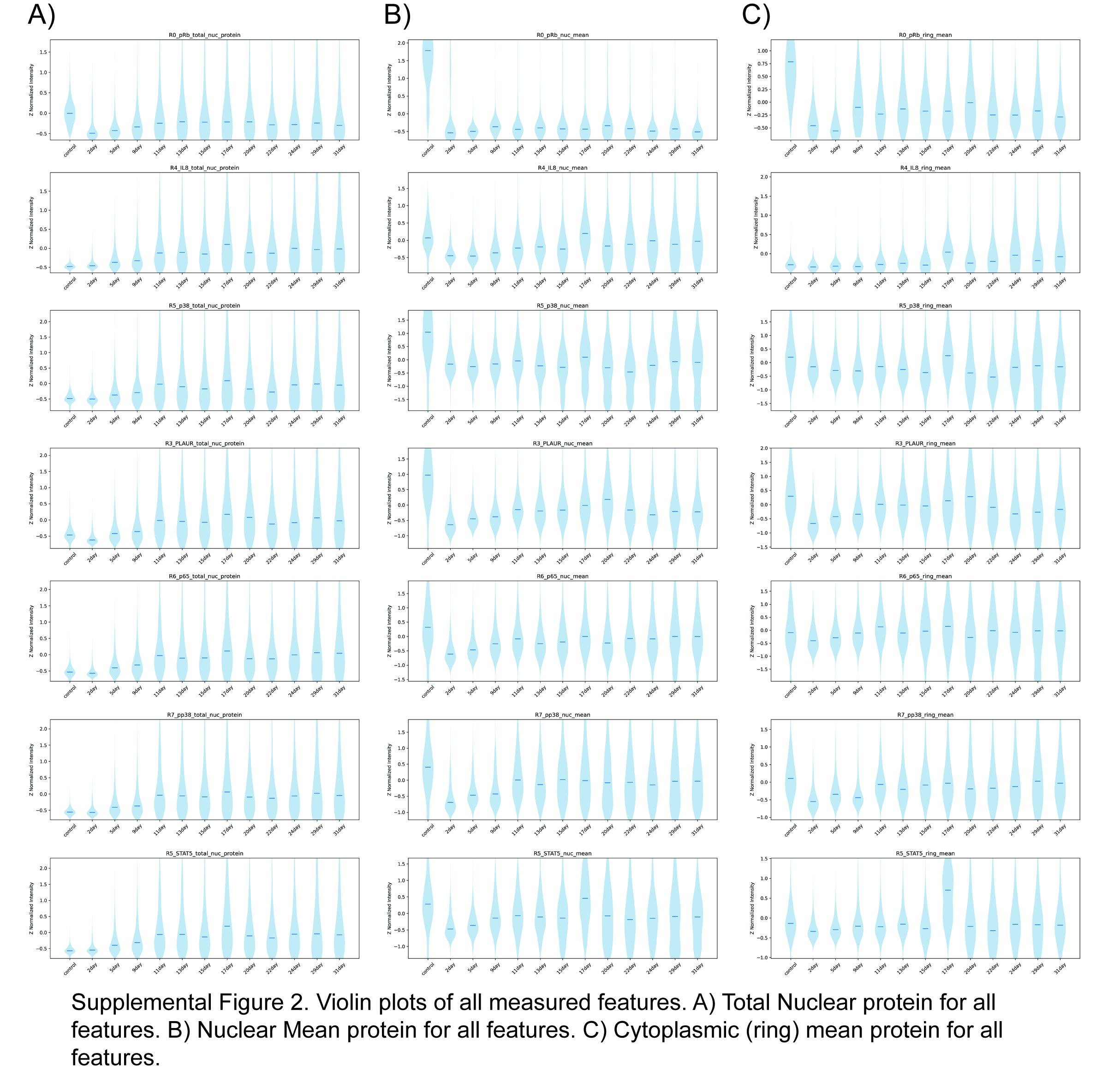

Supplement: Supplementary file 7 — High resolution image (TIF 1000 KB) [file 11357_2024_1503_MOESM4_ESM.tif]

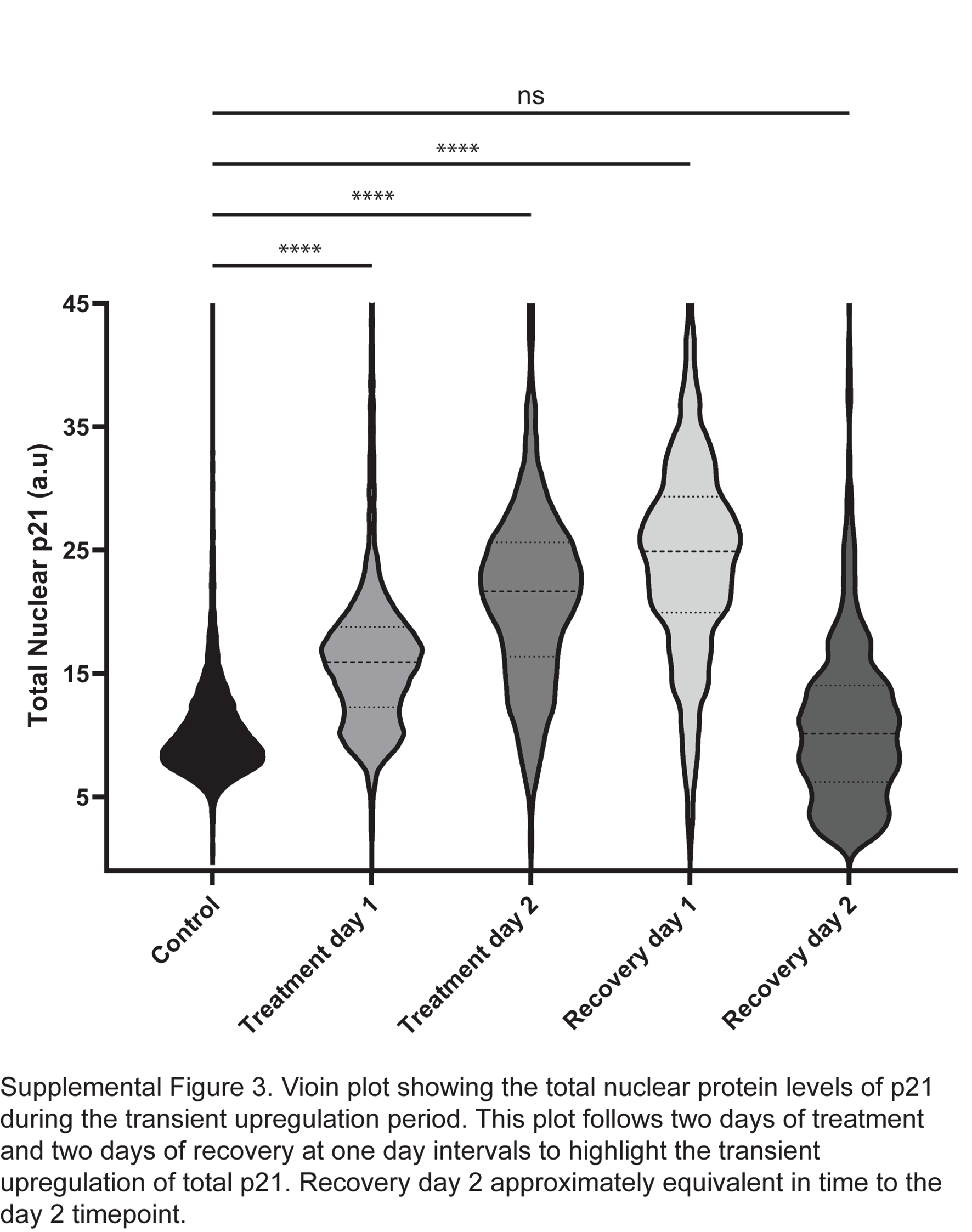

Supplement: Supplementary file 8 — Supplementary Fig. 3 [file 11357_2024_1503_Fig11_ESM.png]

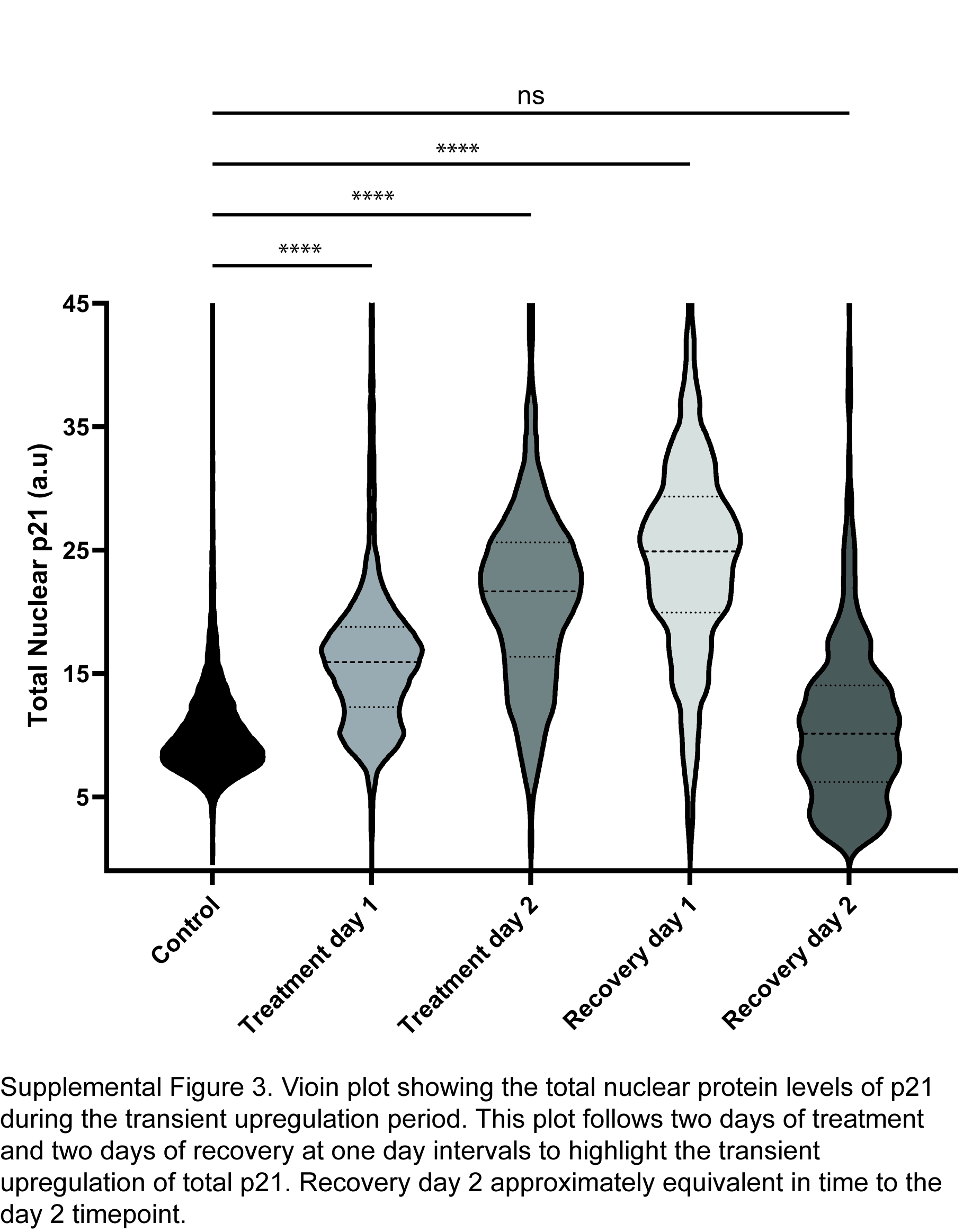

Supplement: Supplementary file 9 — High resolution image (TIF 3698 KB) [file 11357_2024_1503_MOESM5_ESM.tif]

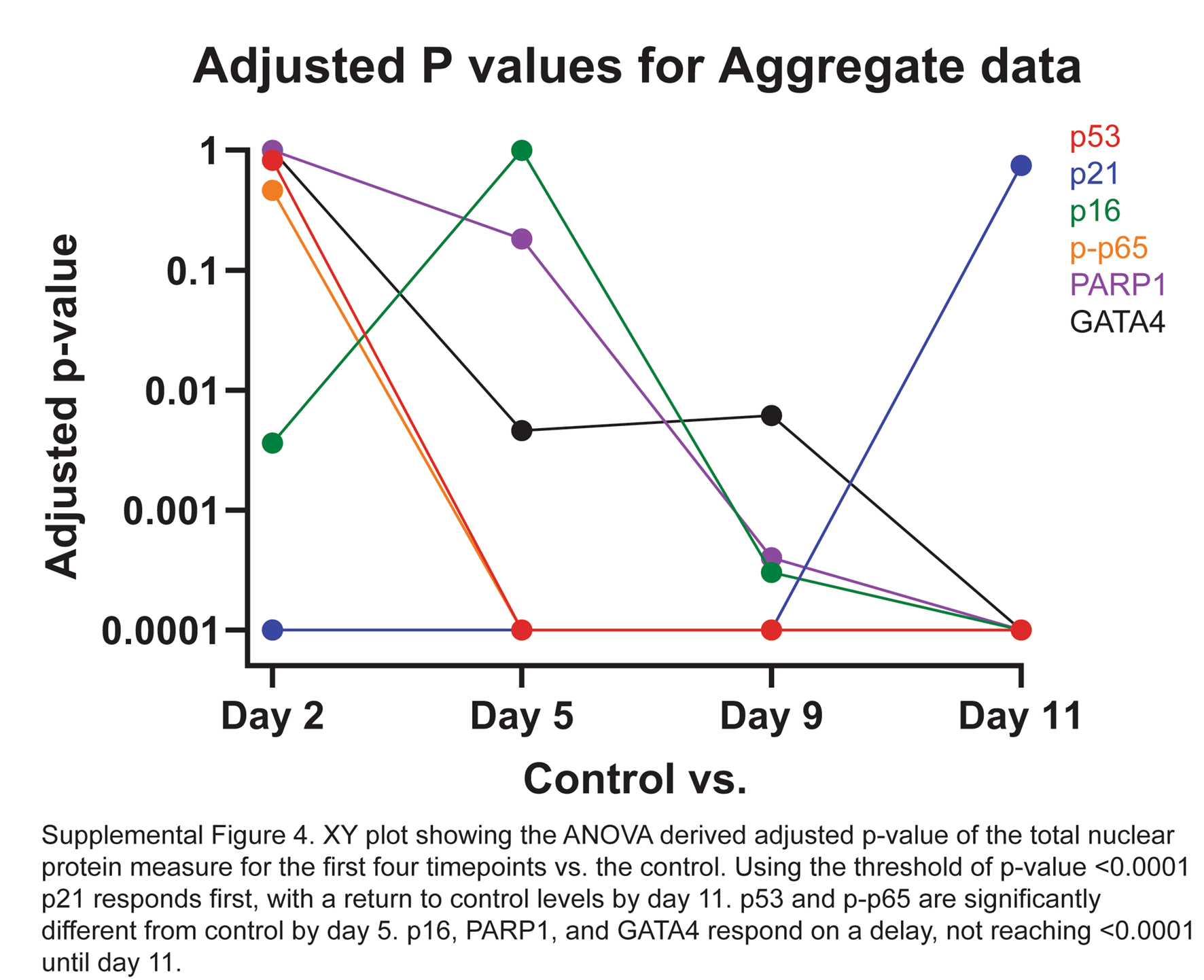

Supplement: Supplementary file 10 — Supplementary Fig. 3 [file 11357_2024_1503_Fig12_ESM.png]

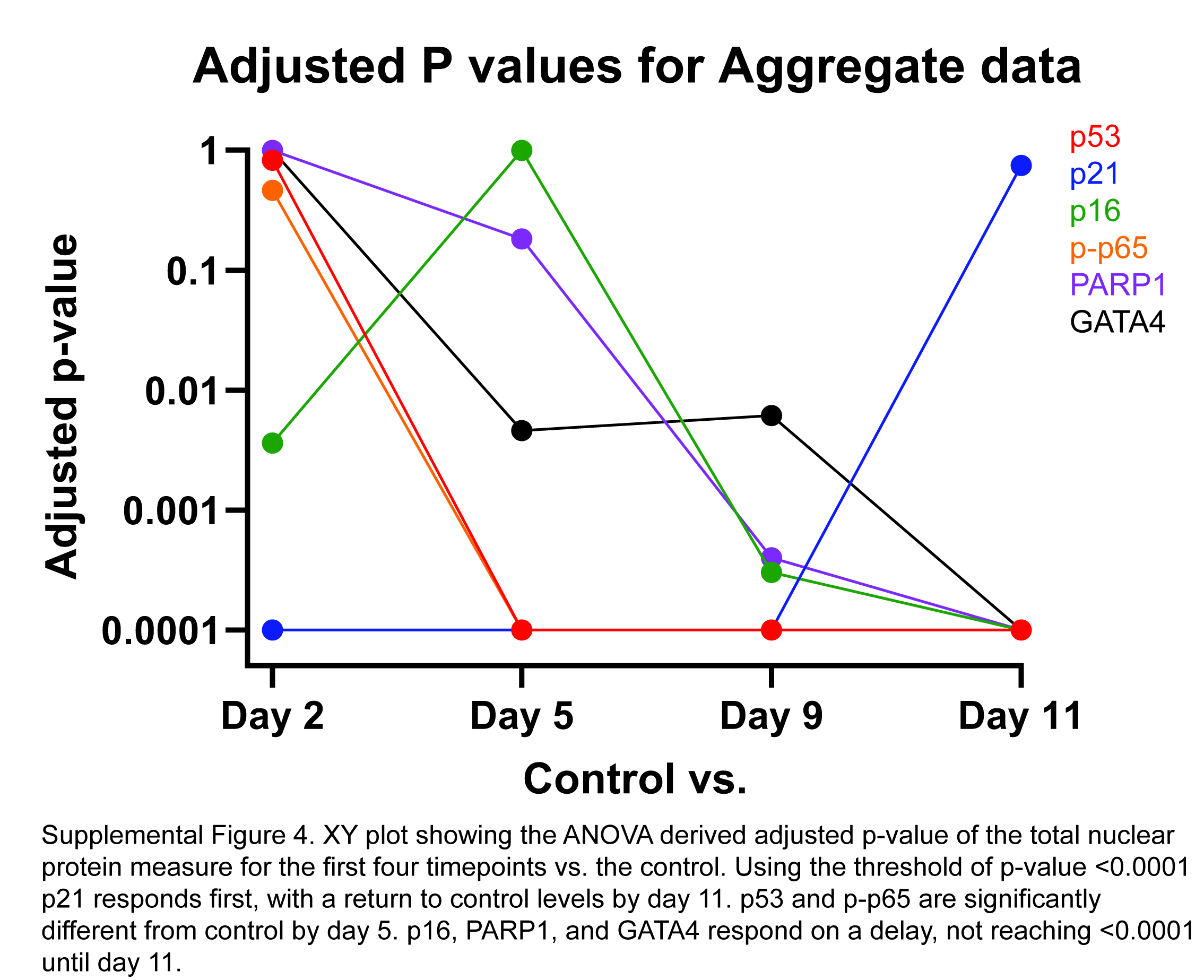

Supplement: Supplementary file 11 — High resolution image (TIF 4357 KB) [file 11357_2024_1503_MOESM6_ESM.tif]
